# Development of tropoelastin-functionalized anisotropic PCL scaffolds for musculoskeletal tissue engineering

**DOI:** 10.1093/rb/rbac087

**Published:** 2022-10-27

**Authors:** Miao Zhang, Ziyu Wang, Anyu Zhang, Linyang Liu, Suzanne M Mithieux, Marcela M M Bilek, Anthony S Weiss

**Affiliations:** Charles Perkins Centre, The University of Sydney, Sydney, NSW 2006, Australia; School of Life and Environmental Sciences, The University of Sydney, Sydney, NSW 2006, Australia; Charles Perkins Centre, The University of Sydney, Sydney, NSW 2006, Australia; School of Life and Environmental Sciences, The University of Sydney, Sydney, NSW 2006, Australia; Applied and Plasma Physics Laboratory, School of Physics, The University of Sydney, Sydney, NSW 2006, Australia; School of Biomedical Engineering, The University of Sydney, Sydney, NSW 2006, Australia; Charles Perkins Centre, The University of Sydney, Sydney, NSW 2006, Australia; School of Life and Environmental Sciences, The University of Sydney, Sydney, NSW 2006, Australia; Charles Perkins Centre, The University of Sydney, Sydney, NSW 2006, Australia; School of Life and Environmental Sciences, The University of Sydney, Sydney, NSW 2006, Australia; Applied and Plasma Physics Laboratory, School of Physics, The University of Sydney, Sydney, NSW 2006, Australia; School of Biomedical Engineering, The University of Sydney, Sydney, NSW 2006, Australia; Charles Perkins Centre, The University of Sydney, Sydney, NSW 2006, Australia; School of Life and Environmental Sciences, The University of Sydney, Sydney, NSW 2006, Australia; Sydney Nano Institute, The University of Sydney, Sydney, NSW 2006, Australia

**Keywords:** tropoelastin, PCL, musculoskeletal tissue engineering, plasma immersion ion implantation, electrospinning, anisotropy, hierarchical

## Abstract

The highly organized extracellular matrix (ECM) of musculoskeletal tissues, encompassing tendons, ligaments and muscles, is structurally anisotropic, hierarchical and multi-compartmental. These features collectively contribute to their unique function. Previous studies have investigated the effect of tissue-engineered scaffold anisotropy on cell morphology and organization for musculoskeletal tissue repair and regeneration, but the hierarchical arrangement of ECM and compartmentalization are not typically replicated. Here, we present a method for multi-compartmental scaffold design that allows for physical mimicry of the spatial architecture of musculoskeletal tissue in regenerative medicine. This design is based on an ECM-inspired macromolecule scaffold. Polycaprolactone (PCL) scaffolds were fabricated with aligned fibers by electrospinning and mechanical stretching, and then surface-functionalized with the cell-supporting ECM protein molecule, tropoelastin (TE). TE was attached using two alternative methods that allowed for either physisorption or covalent attachment, where the latter was achieved by plasma ion immersion implantation (PIII). Aligned fibers stimulated cell elongation and improved cell alignment, in contrast to randomly oriented fibers. TE coatings bound by physisorption or covalently following 200 s PIII treatment promoted fibroblast proliferation. This represents the first cytocompatibility assessment of novel PIII-treated TE-coated PCL scaffolds. To demonstrate their versatility, these 2D anisotropic PCL scaffolds were assembled into 3D hierarchical constructs with an internally compartmentalized structure to mimic the structure of musculoskeletal tissue.

## Graphical Abstract

**Figure:**
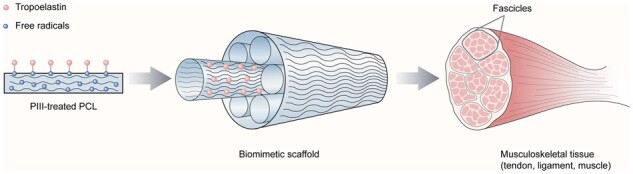

## Introduction

An ability to replicate tissue structure while promoting cell interactions is critical for the design and fabrication of functional constructs in order to repair and replace damaged tissues and organs [1]. In musculoskeletal tissues such as tendon, ligament and muscle, their inherent structural anisotropy, complex fibrous hierarchical architecture and multi-compartmental organization provide a challenge to tissue engineering, yet these features are needed for full functionality [2, 3]. In tendon, the longitudinal alignment of increasingly complex fiber bundles (encompassing subfascicle, fascicle and tertiary fiber bundle components) contributes to a blend of cell interactions and robust tensile strength [4]. While the presence of multiple fiber bundles, covered by successive connective tissue sheaths of endotenon, epitenon and peritenon, protect tendon from damage by limiting the spread of defects and further enhance structural strength [5]. In addition, sliding between and within the fiber bundle compartments allows for effective and efficient transmission of tension despite continual changes in angle and shape during movement [6, 7]. In combination, this intricate architecture allows tendons to form a conduit between muscle and bone that can transmit tensile force effectively and stabilize the joint during body locomotion, but presents a challenge to tissue engineering seeking to replicate layers of complexity using biologically compatible materials, while promoting cell interactions [1].

In an attempt to mimic these features and produce functionally relevant scaffolds, diverse approaches have been used to generate anisotropic structures to provide temporary mechanical support at defect sites and stimulate neotissue growth [8, 9]. However, critically, such engineered constructs typically lack the complex hierarchical arrangement and compartmentalization to direct growth intended for long-term function and stability. There is an unmet demand for new methods to generate such hierarchically structured scaffolds using anisotropic design principles with biodegradable and benign components.

Here, we present a method for the fabrication of an aligned microfibrous polycaprolactone (PCL) scaffold using a combination of electrospinning and mechanical stretching, with introduced anisotropy. Furthermore, to enhance cell-interactive properties, the scaffold was surface functionalized with the extracellular matrix (ECM) protein tropoelastin (TE). TE has been widely investigated as a bioactive molecule that can promote cell adhesion, migration and proliferation and has been shown to encourage the regeneration of diverse tissues including skin, cartilage, nerve and blood vessels [10–14]. We tested physisorption and plasma ion immersion implantation (PIII) as alternative approaches to molecularly coat the scaffolds with TE. Physisorbed proteins bind to the polymeric surface non-specifically with weak non-covalent interactions, making them susceptible to Vroman replacement by other proteins with stronger affinities after implantation [11, 15–17]. In contrast, PIII utilizes energetic ion-assisted plasma modification to generate long-lived free radicals at the surface of a polymeric scaffold, allowing for more stable covalent immobilization of a uniform protein monolayer conveniently without the need for cross-linking chemicals [17–19]. Following coating, TE on the scaffolds was confirmed by Fourier-transform infrared (FTIR) spectroscopy. PIII treatment up to 800 s did not alter the scaffold morphology at a microscale while shorter PIII treatment times of 200 s allowed for covalent attachment that promoted fibroblast proliferation. This effect compared with that of physisorbed TE. The resulting fiber-aligned cell-supporting scaffolds were then further assembled into 3D multi-compartmental hierarchical constructs. These structures are versatile and can be fabricated to imitate tendon organization. By providing relevant topographical and biochemical cues, the use of these components provides a platform technology for the generation of diverse hybrid PCL/TE constructs.

## Materials and methods

### Fabrication of electrospun PCL scaffolds

PCL (average molecular weight = 80 g/mol, Sigma-Aldrich) was dissolved in 1,1,1,3,3,3-hexafluoro-2-propanol (Sigma-Aldrich) at 10% w/v for electrospinning. The solution was loaded into a 1-ml syringe and passed through a steel 18-gauge needle at a constant flow rate of 3 ml/hr. A positive charge of +7.5 kV was applied to the steel needle and a negative charge of −7.5 kV was applied to the collector. The 40-mm diameter circular, aluminum collector was placed at a tip-to-collector distance of 20 cm. For each scaffold, 0.4 ml of the solution was used. The humidity during electrospinning was strictly controlled to 73–76% to enable fabrication of homogenous PCL films.

### Mechanical stretching

**Figure 1.**
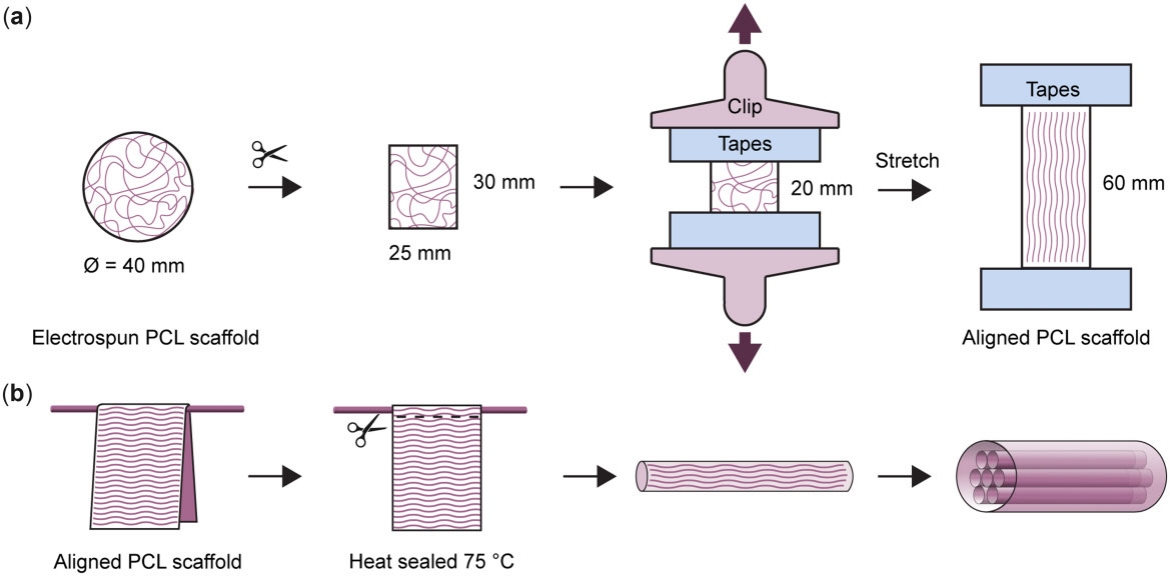
Fabrication of electrospun PCL scaffold with aligned fibers and 3D hierarchical structure. (**a**) Schematic diagram showing the fabrication of electrospun PCL scaffolds with aligned fibers via mechanical stretching. (**b**) Aligned PCL scaffolds were rolled into hollow cylinders with various diameters and heat-sealed. The hollow cylinders were assembled to form a hierarchical structure that approximated native tendon.

Mechanical stretching was used to enhance the fiber alignment of the electrospun PCL scaffolds. The scaffolds were cut into 30×25 mm rectangles (Fig. 1a). A load of 13 kN was applied to the bottom to stretch the scaffold. When the scaffold reached an elongation of 60 mm, the load was allowed to rest on a rack to stop further stretching and the stretch was maintained for 3 hr.

### Scanning electron microscopy

Scaffolds were sputter-coated with 5 nm of gold and the surface morphologies of the scaffolds were imaged with a Zeiss Sigma HD scanning electron microscope (SEM, Zeiss) using an accelerating voltage of 10 kV in secondary electron mode.

### Characterization of the PCL scaffolds

ImageJ (National Institutes of Health) was used to measure fiber diameter, fiber alignment, pore sizes and porosity of the scaffolds from SEM images. Three fields of view (FOV) were imaged for each sample. To determine average fiber diameter, a line was drawn diagonally across a FOV image and the thickness of the first 15 fibers to cross the line was recorded. Fiber alignment was measured [20, 21] using grayscale 8-bit SEM images (×1000) that were inverted before processing for fast Fourier transform (FFT). The Oval Profile plugin (authored by Bill O’Connell, https://imagej.nih.gov/ij/plugins/oval-profile.html) was applied to the generated FFT image and a radial sum intensity plot of 360 angles was generated. The angle of interest was represented by the angle with the highest peak of radial sum intensity. The orthogonal angle, opposite orthogonal angle and opposite angle of interest were calculated according to the following equations:

orthogonal angle=angle of interest+90°, angle of interest<180°angle of interest-90°, angle of interest≥180°,

opposite orthogonal angle=orthogonal angle+180°, orthogonal angle<180°orthogonal angle-180°, orthogonal angle≥180°,

opposite angle of interest=angle of interest+180°, angle of interest<180°angle of interest-180°, angle of interest≥180°.

The radial sum intensities of 10 angles, 1° apart, around each forementioned angle were considered along with the angle itself. The ratio to the mean orthogonal of the angle of interest was calculated according to the following equations:

$$\mathrm{RMS}=\;\sqrt{\sum_{i=1}^{360}\frac{A_{i}^{2}}{360},}$$

$${\mathrm{Ave}}_{M}=\frac{1}{22}\times \sum_{j=1}^{22}\frac{M_{j}}{\mathrm{RMS}},$$

$$\mathrm{Ratio}\;\mathrm{to}\;\mathrm{the}\;\mathrm{mean}\;\mathrm{orthogonal}\;\mathrm{angle}=\;\left[\left(\frac{1}{22}\times \sum_{k=1}^{22}\frac{N_{k}}{\mathrm{RMS}\times {\mathrm{Ave}}_{M}}\right)\times 100\right]-100,$$

where

RMS = root mean square of all radial sum intensities.

*A* = the radial sum intensity of all 360 angles.

*M* = the radial sum intensities of 22 angles, including the orthogonal angle ±5° flanking angles and the opposite orthogonal angle ±5° flanking angles.

*N* = the radial sum intensities of 22 angles, including the angle of interest ±5° flanking angles and the opposite angle of interest ±5° flanking angles.

The extent of fiber alignment was quantified by the numeric value of the ratio to the mean orthogonal angle, where a large value indicates more fibers are oriented in one direction. The pores were identified and measured using the function ‘Analyse Particle’, with a threshold of 10 pixels. The pore size was measured as minimum Feret’s diameter.

### TE surface immobilization by either physisorption or PIII

For PIII treatment, scaffolds were mounted onto a substrate holder and placed 45 mm behind an electrically biased mesh. PIII treatment was performed in a vacuum chamber with nitrogen plasma generated by radio frequency (RF) power at 13.56 MHz. The forward power was 100 W and the reverse power was 12 W. Plasma ions were accelerated by applying negative high voltage (−20 kV) bias pulses of 20 µs duration at a frequency of 50 Hz and drawing a current of 1.1–1.3 mA to the biased substrate holder and its mesh. The base pressure of the vacuum system was 5 × 10^−5 ^Torr and the pressure of nitrogen during the implantation was 2.0 × 10^−3 ^Torr. The PIII treatment was carried out on one side of the scaffold for durations of 200, 400 and 800 s.

Human recombinant TE (Weiss Laboratory) was dissolved in PBS at 20 µg/ml overnight at 4°C. Untreated samples and PIII-treated samples were incubated in 20 µg/ml TE solution for 16 hr at 4°C to facilitate immobilization of the protein through physisorption or covalent bonding, respectively. The samples were washed three times with PBS to remove excess protein.

### FTIR spectroscopy by attenuated total reflectance

FTIR spectroscopy in attenuated total reflectance (FTIR-ATR) mode was performed using a VERTEX 70v FT-IR Spectrometer (Bruker) to examine the surface chemistry of the untreated and PIII-treated scaffolds with and without TE coating. Before FTIR-ATR analysis, TE-coated PCL was washed with 1 ml 2% SDS in Milli-Q water for 1 hr at 40°C to remove loosely bound protein, followed by washing three times with 1 ml Milli-Q water [22]. Each spectrum was collected by averaging a total of 1000 scans at a spectral resolution of 4 cm^−1^ over 4000–700 cm^−1^. Spectral analysis was performed with OPUS software (Bruker). Prior to analysis, a spectrum of atmospheric water and CO_2_ was subtracted from the sample spectra to eliminate the background signal. The absorbance due to vibrations of amide bond in the protein backbone was used to detect the presence of TE. The visibility of these vibrations in the spectra was enhanced by subtracting a PCL-only scaffold spectrum from the TE-functionalized scaffold spectra. Covalent bonding of the TE to the surfaces was detected by its resistance to removal by the hot SDS wash.

### Cell culture and maintenance

Human dermal neonatal fibroblasts (NHF45C, ThermoFisher) were grown in Dulbecco’s Modified Eagle’s Medium (Life Technologies) supplemented with 10% (v/v) fetal bovine serum (Life Technologies) and 1% (v/v) penicillin–streptomycin (Life Technologies) and were used at passages 8–10. Cells were incubated in T75 culture flasks at 37°C and 5% CO_2_ and the media were changed every second day.

### Cell culture on PCL scaffolds

Prior to cell culture, scaffolds were sterilized using UV irradiation for 30 min per side. They were then cut into 10 mm diameter of circular scaffolds, under aseptic conditions and placed at the bottom of a 48-well cell culture plate and secured in place with a glass holder. Human neonatal dermal fibroblasts were then seeded on the surface of the scaffold at a cell density of 5000 cells/cm^2^ and cultured at 37°C, 5% CO_2_ for 7 days_._ Media were changed every other day.

### Confocal microscopy for cell proliferation and morphology analysis

For confocal microscopy, samples with cells cultured on scaffolds were fixed in 4% paraformaldehyde (Sigma-Aldrich) for 15 min at room temperature. Cells were stained with rhodamine phalloidin (ActinRed 555 ReadyProbes, Invitrogen) for 30 min, followed by 5 μg/ml Hoechst 33342 (Invitrogen) for 1 hr at room temperature. Samples were then mounted onto glass slides with Fluoromount-G mounting medium (Thermofisher) and coverslipped. Cells were visualized with a Nikon C2 Confocal Microscope using laser excitation at 405 nm (cell nuclei) and 561 nm (actin). For each sample, five FOVs were imaged in Z-stacks and converted to maximum projection images for further analysis.

To study the cell morphology and proliferation on the scaffolds, confocal images obtained on days 1, 4 and 7 post-seeding were analyzed using ImageJ. Cell numbers on Day 7 were measured by counting Hoechst-positive nuclei using the Analyse Particle plugin. The color threshold was manually adjusted to exclude background noise on each image. The nuclear aspect ratio, which represents the ratio between the major and minor axis of the nucleus, was measured to quantify cellular elongation. To quantify cell alignment on the scaffolds, their actin alignment was measured as described for scaffold fiber alignment.

### Assembly of hierarchical 3D PCL scaffolds

To assemble hierarchical 3D scaffolds, single-walled cylinders were first fabricated by rolling aligned PCL scaffolds on metal rods with diameters of 0.75 or 3 mm (Fig. 1b) and heat-sealing them using a preheated clip at 75°C. The aligned fibers were positioned parallel to the longitudinal axis of the rod. Six 0.75 mm diameter PCL cylinders were then inserted into a larger 3 mm diameter cylinder to form a 3D scaffold with an aligned, multi-compartmental structure.

### Statistical analysis

Data are expressed as mean ± standard deviation. Statistical analyses used GraphPad Prism (version 8). Student’s *t*-tests were performed to analyze fiber characteristics and cell morphology on unstretched and stretched PCL scaffolds, while two-way analysis of variance (ANOVA) with Dunnett’s T3 multiple comparison tests were used for scaffold characterization analysis after PIII. Ordinary one-way ANOVA with Tukey multiple comparison was performed for cell proliferation. Significant difference is indicated in figures as *P ≤ 0.05, **P≤ 0.01, ***P ≤0.001 and ****P ≤0.0001.

## Results

### Fabrication of PCL scaffolds with random and aligned fibers

**Figure 2.**
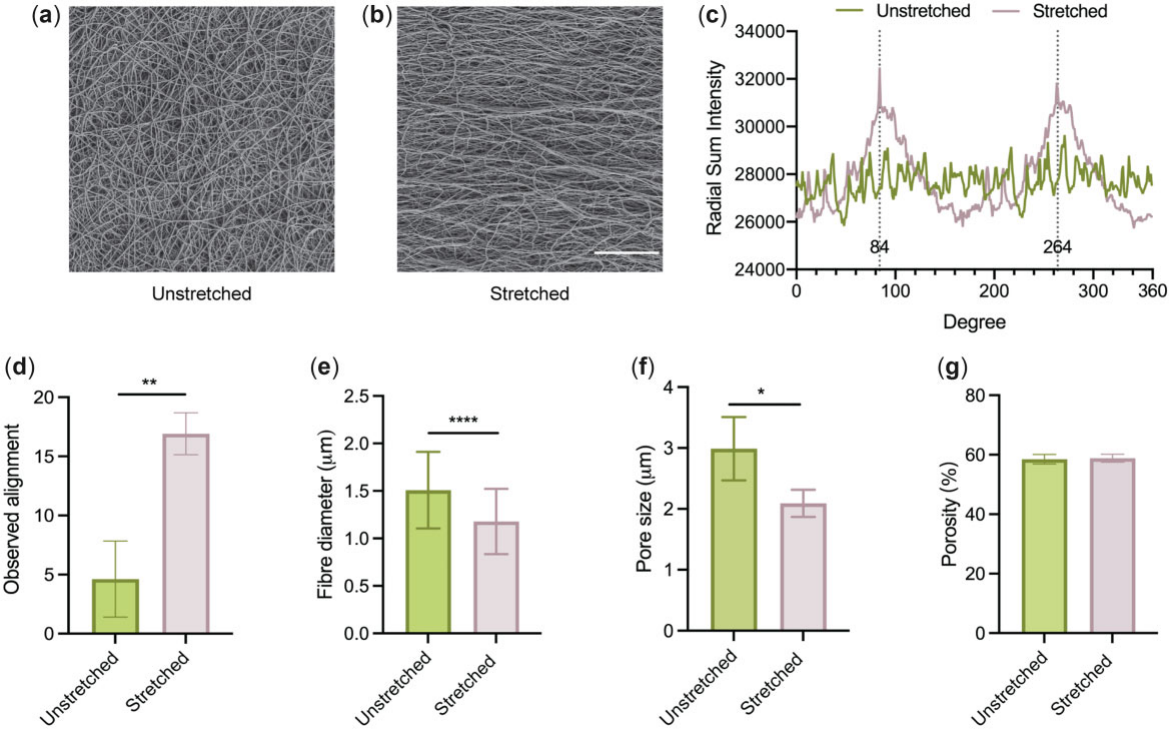
Morphological characterization of the unstretched and stretched scaffolds. SEM image of an (**a**) unstretched and (**b**) stretched scaffold. Scale bar = 100 µm. Brightness and contrast have been adjusted for clarity. (**c**) Radial sum intensity analysis of unstretched and stretched PCL scaffolds. (**d**) Observed alignment analysis of unstretched and stretched PCL scaffolds. (**e**) Fiber diameter of unstretched and stretched scaffolds. *n* = 180. (**f**) Pore sizes within PCL scaffolds were reduced following mechanical stretching while (**g**) porosity remained unchanged. *n* = 4.

Electrospun PCL scaffolds showed randomly oriented PCL fibers with an average diameter of 1.5 ± 0.4 µm (Fig. 2a). Stretching the PCL scaffolds with a constant load until they doubled in length resulted in final dimensions of 51.5 ± 2.4 × 16.8 ± 0.8 mm (*n* = 30). SEM images of the stretched scaffolds revealed wave-like fibers primarily aligned in the direction of stretch (Fig. 2b). Analysis of fiber direction, using a radial sum intensity plot showed two distinct peaks 180° apart for the stretched scaffolds, which indicated the majority of fibers were aligned (Fig. 2c); in contrast, no distinct peaks were observed for unstretched scaffolds, confirming that the fibers were randomly oriented. The alignment of fibers was further confirmed using their ratio to the mean orthogonal angle. This was significantly larger in stretched scaffolds compared with unstretched scaffolds, indicating an enhancement in fiber alignment after mechanical stretching (Fig. 2d).

Mechanical stretching was used to adjust the scaffold fiber diameter and pore size. Unstretched fibers had an average diameter of 1.5 ± 0.4 μm while the stretched fibers had a significantly thinner average diameter of 1.2 ± 0.4 μm (Fig. 2e). Pore sizes in these microfibrous scaffolds significantly decreased from 3.0 ± 0.5 to 2.1± 0.2 μm post-stretching (Fig. 2f) while the porosity of the scaffolds was maintained at 58% before and after mechanical stretching (Fig. 2g).

### Mechanically stretched scaffolds improve cell alignment

**Figure 3.**
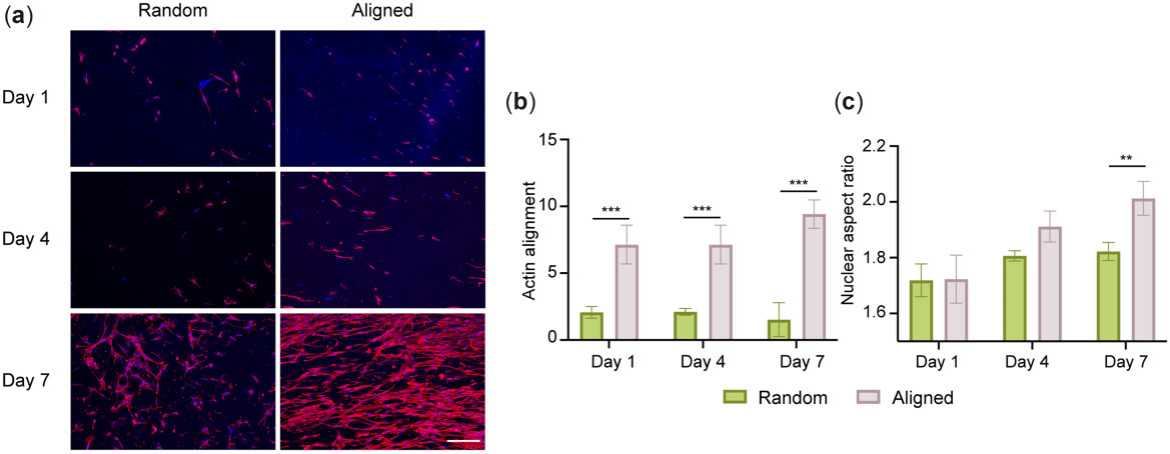
Aligned fibers induce cell elongation and alignment. (**a**) Representative confocal images of fibroblasts seeded on PCL scaffolds and stained for actin (red) and nuclei (blue). Scale bar = 200 µm. Brightness and contrast have been adjusted for clarity. (**b**) Observed actin alignment of fibroblasts seeded on random and aligned PCL scaffolds. (**c**) Nuclear aspect ratio of fibroblasts seeded on random and aligned PCL scaffolds. For each group, *n* = 3.

Fibroblasts were cultured on PCL scaffolds with either randomly oriented or aligned fibers for 7 days and confocal microscopy of stained actin filaments was used to quantitatively compare cell morphologies (Fig. 3a). Human dermal fibroblasts were used in this pilot study as sufficient human tendon-specific cells are extremely difficult to source and previous studies have demonstrated that the *in vivo* implantation of fibroblast-laden cell-scaffolds produced neo-tendons that had similar mechanical properties, collagen alignment and fibril diameter to neo-tendons derived from tenocyte-laden cell-scaffold implants in both murine and porcine tendon defect model surgeries [23, 24]. On randomly oriented fibers, fibroblasts displayed cytoskeletons that extended in multiple directions with an arbitrary cell orientation. In contrast, on aligned fibers the fibroblasts responded to contact guidance with extended actin filaments that aligned along the longitudinal axis of the fibers (Fig. 3b). The cell nuclei were correspondingly elongated, as indicated by their increased nuclear aspect ratio by day 7 (Fig. 3c).

### Scaffolds were coated with TE by either physisorption or after PIII treatment

**Figure 4.**
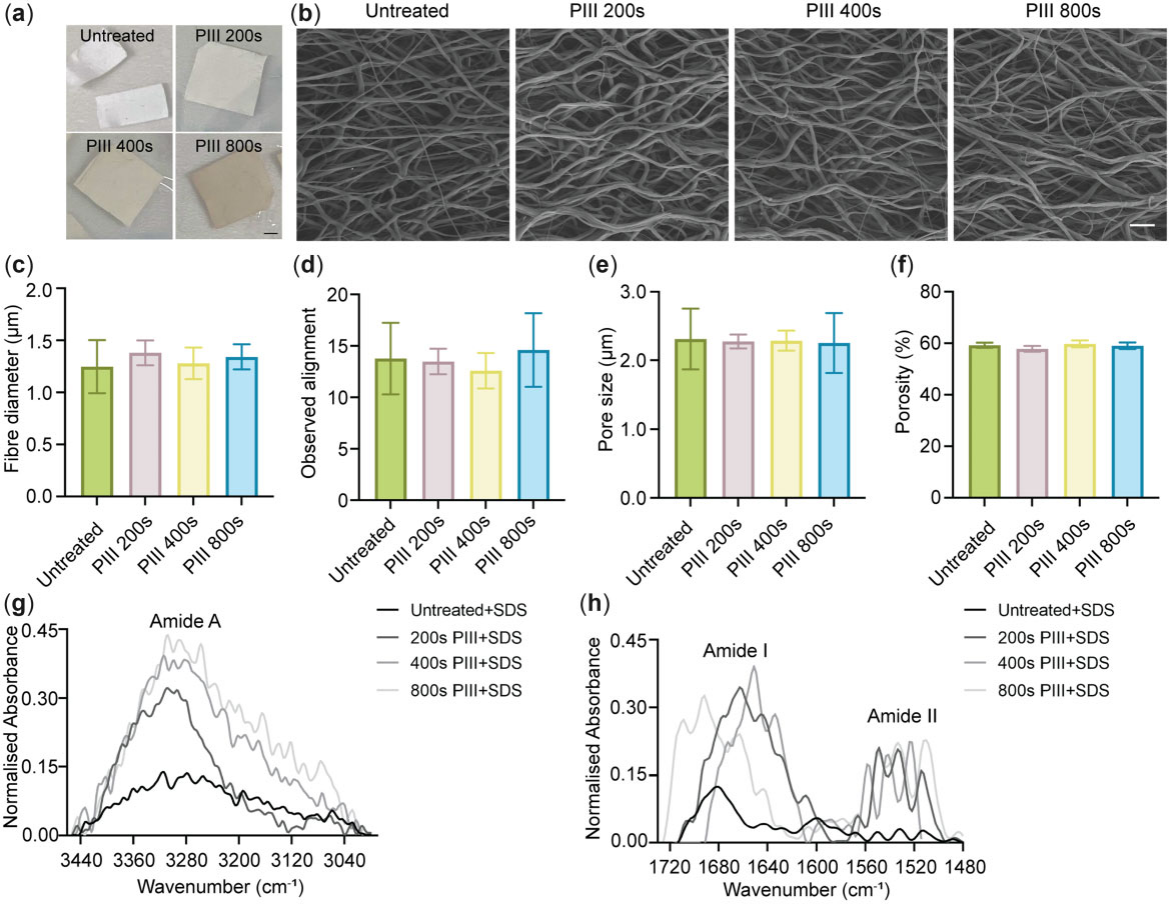
Characterization of PIII-treated scaffolds. (**a**) PIII treatment induced a darkening of the scaffold surface. Scale bar = 1 cm. (**b**) SEM micrograph of stretched PCL scaffolds after various durations of PIII treatment. Scale bar = 10 µm. PIII treatment did not alter the (**c**) fiber diameter, (**d**) fiber alignment, (**e**) pore size and (**f**) porosity of the stretched PCL scaffolds. *n* = 6. (**g** and **h)** Attached TE detected using FTIR-ATR spectra of PIII-treated and untreated samples.

For covalent attachment of TE, the scaffolds were PIII treated for 200–800 s. PIII treatment resulted in a visual darkening of the PCL scaffold surfaces. This intensified with increased treatment time (Fig. 4a), owing to the formation of carbonized clusters [22]. However, the morphologies of the stretched PCL scaffolds did not change with PIII treatment up to 800 s. This persistence was evidenced by no significant change in fiber diameter and alignment, pore size or porosity (Fig. 4b–f) [22]. Untreated and PIII-treated PCL scaffolds were surface-functionalized with TE by incubating them in 20 µg/ml TE solution. For FTIR analysis, the scaffolds were then washed in SDS to remove non-covalently attached TE [22]. A persistence of covalently attached TE was confirmed by FTIR-ATR analysis, which detected the presence of Amide A (3300 cm^−1^), Amide I (1720–1580 cm^−1^) and Amide II peaks (1575–1475 cm^−1^) in PIII-treated PCL scaffolds (Fig. 4g and h). In contrast, the untreated scaffolds did not display obvious peaks, indicating that physisorbed TE had been removed by SDS washing [18].

### Cell proliferation on TE functionalized PCL scaffolds

**Figure 5.**
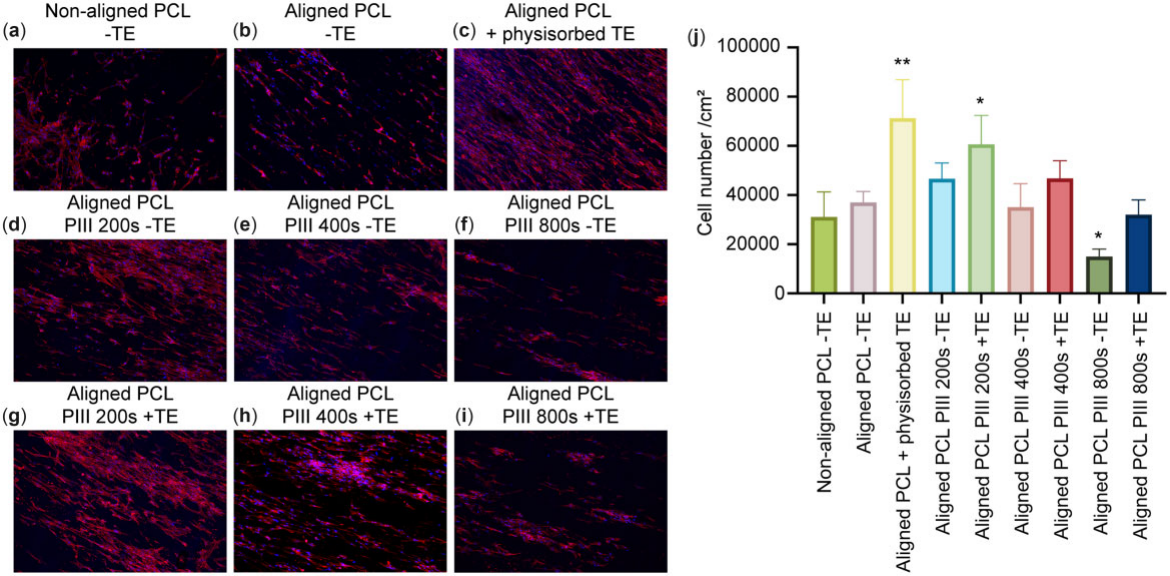
Representative confocal images of fibroblasts seeded on (**a**) non-aligned PCL scaffold and (**b**–**i**) aligned PCL scaffolds, with and without TE, stained for actin (red) and nuclei (blue). Scale bar = 200 μm. Brightness and contrast have been adjusted for clarity. (**j**) Fibroblast numbers (per cm^2^) on PCL scaffolds at day 7. Scaffolds were initially seeded with 5000 cells/cm^2^. * represents comparison with aligned PCL-TE. For each group, *n* = 3.

Cell growth on aligned fibers with TE functionalization was assessed by culturing fibroblasts on the PCL scaffolds for up to 7 days (Fig. 5a–i). The orientation of PCL fibers did not alter fibroblast proliferation (Fig. 5j). Physisorbed TE, and covalently bound TE facilitated through 200 s PIII treatment, significantly promoted cell proliferation compared with uncoated aligned PCL fiber scaffolds (PCL-TE). This was consistent with previous studies showing that TE enhances cell proliferation [18]. In contrast, cell proliferation was not stimulated after longer PIII treatment times.

### Hierarchical and compartmentalized construct fabricated from aligned PCL scaffolds

**Figure 6.**
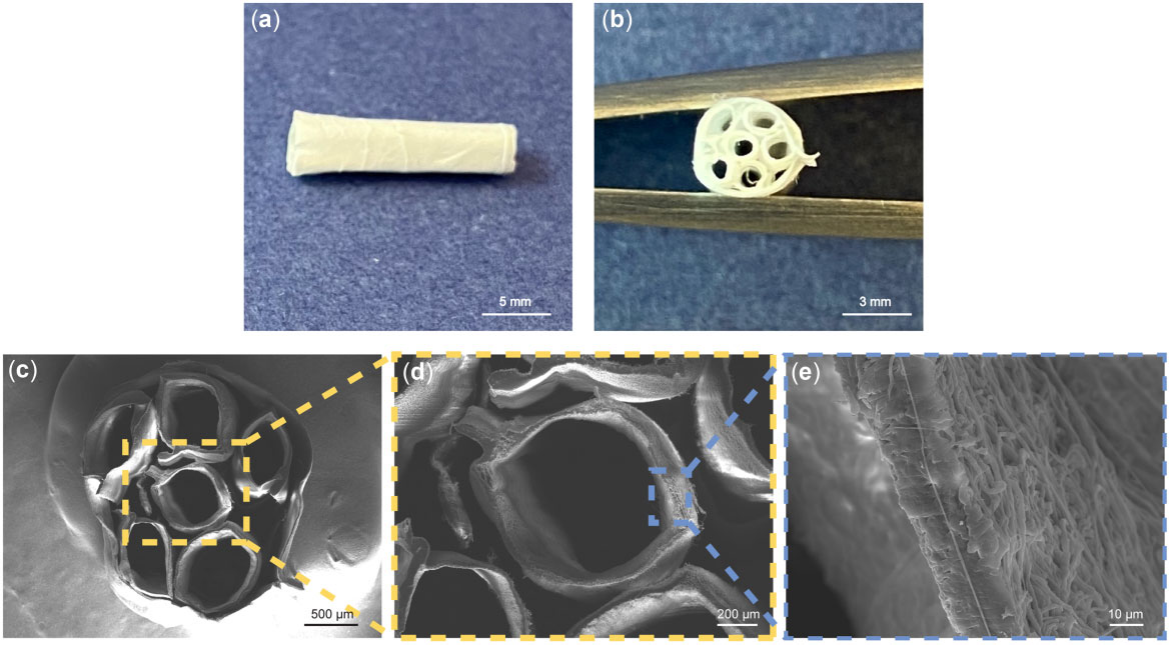
Morphology of 3D tendon scaffold. Longitudinal view (**a**) and a cross-sectional view (**b**) of the 3D scaffold. (**c**) SEM images of the cross-section of the 3D construct. (**d**) Cross-section of the hollow cylinder in the core portion. (**e**) A magnified cross-section view of the hollow cylinder in the core.

PCL scaffolds with aligned fibers were assembled into 3D compartmentalized constructs by rolling and heat-sealing (Fig. 1b). The constructs comprised a core-shell structure with six internal hollow cylinders at diameters of 0.75 mm and an outer shell with a diameter of 3 mm (Fig. 6a and b), as confirmed by SEM (Fig. 6c–e). Large voids were incorporated to allow for TE-mediated cell attachment, proliferation and presumably neotissue *in vivo*. The direction of electrospun fibers on each tubular structure was aligned along the longitudinal axis, in order to mimic the organization of collagen fibers in tendon, but also may be of value in anisotropic musculoskeletal tissue such as ligaments and muscle [3].

## Discussion

The fabrication approach presented in this study combines conventional static electrospinning and mechanical stretching, which is consistent and cost-effective, yet robust in facilitating fiber alignment, in contrast to alternative common approaches [25–27]. The fabrication technique generates wave-like aligned fibers that mimic the crimping morphology of collagen fibrils in tendons and ligaments (Fig. 2b) [28–30]. As a platform technology, the aligned PCL scaffold provides contact guidance for fibroblast alignment and elongation that persist from day 1 of cell culture [31]. The incorporation of anisotropic topographical cues guides the deposition of ECM in an aligned order and has the potential to provide an inductive environment for tissue-specific growth *in vivo*, which is needed for musculoskeletal tissue regeneration [32–35].

TE was used as a bioactive molecule, attached to the anisotropic scaffolds to promote cell proliferation. Surface coating of the scaffolds with TE was achieved through physisorption or PIII treatment. PIII treatment reduces PCL hydrophobicity and produces reactive radicals at the polymer surface which can react with amino acid side chains facilitating covalent binding of protein molecules [36, 37]. TE physisorption and covalent attachment after short PIII treatment promoted comparable enhancement of fibroblast numbers by 7 days. In this system, the cell responses recapitulated effects on the polyurethane copolymer Elast-Eon E2A, polyethersulfone (PES), polylactic acid/poly(lactic-co-glycolic acid) (PLLA-PLGA) and polytetrafluorethylene (PTFE) [11, 18, 38, 39]. A key benefit of covalently immobilized TE over physisorbed TE is that it is not susceptible to dynamic protein exchange in an *in vivo* implantation environment, such as due to the Vroman effect [17].

Cell density decreased with extended PIII treatment duration, which may be due to the highly carbonized, dehydrogenated and crosslinked surface layers [40]. The extent of carbonization increased with longer treatment time as evidenced by increasing visual darkening [22]. This carbonized layer may exhibit distinct properties from the untreated layer, including surface chemistry, wettability, nanotopography and mechanical properties, all of which affect cell-biomaterial interaction [22]. Therefore, the optimal PIII treatment time that benefits cell proliferation is specific to each biomaterial [18, 41]. For example, endothelial cell adhesion and proliferation were enhanced on 800 s PIII-treated silk scaffolds in the absence of surface-functionalized proteins, which was not seen in this study when fibroblasts were seeded on PCL scaffolds PIII treated for 800 s [41]. Another study showed 400 s PIII-treated PES with a covalently immobilized TE coating promoted fibroblast proliferation compared with PES with physisorbed TE [18]. In this study, use of a short PIII treatment of 200 s to immobilize TE on an aligned PCL scaffold supported greater cell proliferation than longer PIII treatment times of 400 and 800 s.

The combination of features seen with our platform helps to demonstrate its versatility. It is appreciated that scaffolds with core-shell structures have the capacity to regenerate musculoskeletal tissues [31, 42–44]. For example, a core-shell tendon construct could direct the regeneration of dense and aligned fibrous tissue, similar to a tendon fascicle [31]. However, such constructs were assembled by rolling only one PCL sheet with aligned fibers in a concentrically multi-layered format and therefore lacked the multicompartmental structure typical of tendons as described here. A core-shell construct comprised of multiple fiber bundles has been used to resemble the hierarchical arrangement of skeletal muscle, however, its cellular interactions were unclear [44]. In contrast, our method fabricates a cell-supporting construct consisting of multiple cylindrical cores with hollow channels that is intended to mimic a complex tissue structure for musculoskeletal use. On this basis, these constructs have the potential to allow for cell infiltration, ECM deposition and eventual tissue regeneration [44, 45].

## Conclusion

This study focused on the design, characterization and *in vitro* evaluation of a PCL scaffold with aligned fiber structure and a surface TE coating for scaled tissue engineering. The alignment of fibers was enhanced by mechanical stretching. Aligned fibers and the use of TE coatings promoted cell proliferation, elongation and cell alignment. The method is versatile and adaptable such that anisotropic PCL scaffolds can be assembled into a range of compartmentalized 3D hierarchical constructs.
